# Upregulation of CRABP1 in human neuroblastoma cells overproducing the Alzheimer-typical Aβ_42 _reduces their differentiation potential

**DOI:** 10.1186/1741-7015-6-38

**Published:** 2008-12-16

**Authors:** Markus Uhrig, Peter Brechlin, Olaf Jahn, Yuri Knyazev, Annette Weninger, Laura Busia, Kamran Honarnejad, Markus Otto, Tobias Hartmann

**Affiliations:** 1 Center for Molecular Biology of the University of Heidelberg (ZMBH), D-69120 Heidelberg, Germany; 2 Institute for Neurodegeneration and Neurobiology, Neurology, Saarland University, D-66421 Homburg/Saar, Germany; 3 Department of Neurodegeneration and Restorative Research, Center for Neurological Medicine, University of Göttingen, D-37073 Göttingen, Germany; 4 DFG Research Center for Molecular Physiology of the Brain (CMPB), D-37073 Göttingen, Germany; 5 Max Planck Institute for Experimental Medicine, Proteomics, D-37075 Göttingen, Germany; 6 German Cancer Research Center (DKFZ), D-69120 Heidelberg, Germany; 7 Department of Neurology, D-89075 Ulm, Germany

## Abstract

**Background:**

Alzheimer's disease (AD) is characterized by neurodegeneration and changes in cellular processes, including neurogenesis. Proteolytic processing of the amyloid precursor protein (APP) plays a central role in AD. Owing to varying APP processing, several β-amyloid peptides (Aβ) are generated. In contrast to the form with 40 amino acids (Aβ_40_), the variant with 42 amino acids (Aβ_42_) is thought to be the pathogenic form triggering the pathological cascade in AD. While total-Aβ effects have been studied extensively, little is known about specific genome-wide effects triggered by Aβ_42 _or Aβ_40 _derived from their direct precursor C99.

**Methods:**

A combined transcriptomics/proteomics analysis was performed to measure the effects of intracellularly generated Aβ peptides in human neuroblastoma cells. Data was validated by real-time polymerase chain reaction (real-time PCR) and a functional validation was carried out using RNA interference.

**Results:**

Here we studied the transcriptomic and proteomic responses to increased or decreased Aβ_42 _and Aβ_40 _levels generated in human neuroblastoma cells. Genome-wide expression profiles (Affymetrix) and proteomic approaches were combined to analyze the cellular response to the changed Aβ_42_- and Aβ_40_-levels. The cells responded to this challenge with significant changes in their expression pattern. We identified several dysregulated genes and proteins, but only the cellular retinoic acid binding protein 1 (CRABP1) was up-regulated exclusively in cells expressing an increased Aβ_42_/Aβ_40 _ratio. This consequently reduced all-trans retinoic acid (RA)-induced differentiation, validated by CRABP1 knock down, which led to recovery of the cellular response to RA treatment and cellular sprouting under physiological RA concentrations. Importantly, this effect was specific to the AD typical increase in the Aβ_42_/Aβ_40 _ratio, whereas a decreased ratio did not result in up-regulation of CRABP1.

**Conclusion:**

We conclude that increasing the Aβ_42_/Aβ_40 _ratio up-regulates CRABP1, which in turn reduces the differentiation potential of the human neuroblastoma cell line SH-SY5Y, but increases cell proliferation. This work might contribute to the better understanding of AD neurogenesis, currently a controversial topic.

## Background

Alzheimer's disease (AD) is a genetically heterogeneous disorder because mutations in multiple genes are involved along with non-genetic factors [1]. The risk may be determined by the effects of numerous loci, some of which may produce only minor contributions. Amyloid precursor protein (APP), presenilin1, presenilin2 and the apolipoprotein E ε4 allele have been associated with AD [2,3]. These genes are assumed to be responsible for approximately 50% of the genetic background of the disease, suggesting that further susceptibility genes exist. Genetic analyses of kindred with AD have pointed to β-amyloid peptides (Aβ) as the initiating molecules in the development of the disease.

Biochemical work on APP processing revealed that pathogenic mutations alter processing in such a way that more Aβ_42 _is produced. Genetic and biochemical data together suggested that Aβ_42 _accumulation was the primary event in the pathogenesis of AD. Aβ_42_, but not the more abundant Aβ_40_, may cause neuronal dysfunction and trigger neurodegeneration *in vivo *[4,5]. APP is cleaved by β-secretase within its ectodomain, resulting in the generation of the C-terminal fragment C99, which is further cleaved by the γ-secretase complex. APP processing results in the release of different peptides. To focus on Aβ, we used the standard construct that maintains APP sorting and the relevant processing events [6,7]. The pathological mechanism of how Aβ_42 _or Aβ_40 _acts is unclear. To elucidate the underlying mechanisms, we used a combined transcriptomic-proteomic approach and utilized APP point mutations to modulate the Aβ_42_/Aβ_40 _ratio. Using a *genome and proteome-wide *approach provided us with the maximum amount of information possible. We identified cellular retinoic acid binding protein 1 (CRABP1) as the exclusive transcript and protein showing strong differential expression as a consequence of an increased Aβ_42_/Aβ_40 _ratio. Accordingly, cells with the increased Aβ_42_/Aβ_40 _ratio showed a reduced ability to differentiate. Remarkably, a decreased Aβ_42_/Aβ_40_ratio did not affect CRABP1 expression. CRABP1 is involved in retinoic acid (RA)-induced differentiation [8-10] and is expected to play a crucial role in neurogenesis [11].

Neurogenesis is reported to be enhanced in the hippocampi [12] of patients with AD [13] where it may produce cells to replace neurons lost in the disease [14]. The effect of AD on neurogenesis has recently been reproduced in a transgenic mouse model [15] in which APP mutations lead to increased incorporation of BrdU and expression of immature neuronal markers in two neuroproliferative regions: the dentate gyrus and the subventricular zone. As neurogenesis is increased in these mice in the absence of neuronal loss, it might be triggered by more subtle disease manifestations, for example the initial accumulation of the Aβ peptide. In transgenic mice, overexpressing familial AD variants of APP and/or PS1 dramatically diminished survival of newborn neurons *4 weeks after birth *[16]. This data hints at an increased neurogenesis in AD, but in contrast to this, also point to early detrimental events shortly after the neurons are born.

## Methods

For details, see the Additional file 1.

### Plasmids

C99 encoding sequences were cloned into a pCEP4 vector (Invitrogen) resulting in the following constructs: pCEP4-spA4ct-DA-WT, pCEP4-spA4ct-DA-I45F and pCEP4-spA4ct-DA-V50F. The plasmid constructs have been described previously [6,7].

### Cell line, cell culture and transfections

Human neuroblastoma SH-SY5Y cells [17,18] were cultured in 50% Minimum Essential Medium (MEM; Sigma) and 50% Nutrient Mixture F-12, HAM (Sigma), supplemented with 10% fetal bovine serum (FBS; PAN), 1% non-essential amino acid solution (Sigma) and 1% L-Glutamin (Sigma), in a humidified atmosphere with 5% CO_2_. We transfected 70% confluent cells with the constructs described previously.

### Preparation of cell lysates and collection of conditioned media

We added 5 ml culture medium to 70% confluent cells in a 10 cm culture dish and conditioned media were collected after 16–48 hours. The conditioned media were centrifuged at 4°C for 1 minute at 13,000 rpm and the supernatants were used for immunoprecipitation of soluble secreted Aβ. Cell lysates were prepared by harvesting and lysing cells on ice in lysis buffer supplemented with Complete^® ^protease inhibitor (Roche).

### Immunoprecipitation

Conditioned media were immunoprecipitated with protein G-Sepharose (Sigma) and the antibodies G2–10 and G2–11. The immunoprecipitated proteins were separated on 12% Tris-Tricine gels.

### Western blotting and antibodies

Western blot analysis was performed as described elsewhere [19]. Briefly, proteins were detected with the antibody W02, specific for residues 1–10 of Aβ.

### Transcriptomics and data analysis

Gene chip analysis was performed according to the Expression Analysis Technical Manual (Affymetrix) with minor modifications: Briefly, total RNA was extracted using the Qiashredder-Kit, RNeasy Midi-columns and the RNase-free DNase set (Qiagen). A total of 20 μg of RNA was reverse transcribed into cDNA by using oligo(dT) primers (Proligo) and the Superscript™ Double-Stranded cDNA Synthesis Kit (Invitrogen). We *in vitro *transcribed 3.3 μl of purified cDNA using the BioArray™ *High Yield*™ RNA Labeling Kit (Enzo Life Sciences). We fragmented 15 μg of purified cRNA using the GeneChip^® ^Eukaryotic Hybridization Control Kit (Affymetrix). We hybridized 15 μg of fragmented cRNA to whole genome HG-U133 A and HG-U133 B oligonucleotide arrays. Chips were washed, stained, scanned and the quality of the created dat-file images was evaluated by using MAS 5.0 and Gene Operating Software GCOS 1.2 (Affymetrix). The quality of each sample was controlled (see Additional file 1). Transcriptomic data was analyzed with MAS 5.0, GCOS 1.2 (both Affymetrix) and Array Assist 3.3 (Stratagene). Chp-files were created by using the PLIER algorithm. *P*-values were calculated from three independent experiments using either a two class unpaired *t*-test or one-way analysis of variance (ANOVA). Further data analysis was performed with Excel (Microsoft). For data normalization, filtering details and data output, see Additional files 1, 2 and 3.

### Quantitative real-time polymerase chain reaction and selection of an endogenous control for normalization

Total RNA, was reverse transcribed into cDNA using random hexamer primers included in the High-Capacity cDNA Archive Kit (Applied Biosystems). This cDNA was amplified and measured by using TaqMan^® ^Gene expression assays (Applied Biosystems). Cycling conditions were 50°C for 2 minutes, 95°C for 10 minutes, followed by 40 cycles of 95°C for 15 seconds and 60°C for 1 minute. Relative quantification was performed with the 2^-Δ^C_T _method. For normalization, an endogenous control was selected out of 10 candidate controls using the TaqMan^® ^Human Endogenous Control Plate (Applied Biosystems).

### Proteomics: two-dimensional difference gel electrophoresis

Briefly, dried cell pellets were solubilized in lysis buffer, centrifuged and supernatant proteins were labeled with Cy3 as well as Cy5, so that each sample was labeled in a dye-switch manner. Cy2 was used as fluorophor for the internal standard. First dimension isoelectric focusing was performed on Immobilized pH-Gradient Gel Strips pH 3–10 Non-Linear (GE Healthcare). Second dimension sodium dodecyl sulfate polyacrylamide gel electrophoresis (SDS-PAGE) was performed on 12.5% isocratic 254 × 200 × 1 mm^3 ^gels [20]. CyDye fluorescence images were acquired on a laser scanner (GE Healthcare) and protein abundance changes were analyzed with the DeCyder™ 3.0 Software (GE Healthcare) [21]. For subsequent mass spectrometry the proteins were stained with colloidal Coomassie Brilliant Blue [22] and protein spots were excised manually.

### Protein identification

Proteins were identified as described recently [23]. Briefly, an automated platform [24] was used to digest the proteins in-gel with trypsin and to prepare the proteolytic peptides for matrix assisted laser desorption ionization time-of-flight mass spectrometry (MALDI-TOF-MS). For each sample a peptide mass fingerprint (PMF) spectrum and fragment ion spectra of up to four selected precursor ions were acquired within the same automated analysis loop using an Ultraflex I mass spectrometer (Bruker Daltonics). Database searches were performed with the Mascot Software 2.0 (Matrix Science). Only proteins represented by at least one peptide sequence above the significance threshold in combination with the presence of at least four peptide masses assigned in the PMF were considered as identified.

### RNA interference

siRNAs were double-stranded [25,26] pre-designed, annealed Silencer™ siRNAs (Ambion). To transiently knock down *CRABP1*, 30 nM siRNA was used. After 48 hours following transfection with siRNA, total RNA was extracted from the cells and the extent of knock down was measured by real-time polymerase chain reaction (real-time PCR).

### Differentiation assay

SH-SY5Y cells were treated with 0.1–1000 nM RA, in the absence or presence of serum for 2–10 days. Differentiation was evaluated by checking the length, shape and number of outgrowing protrusions by phase contrast microscopy at appropriate times. Phase contrast pictures were taken from living neuroblastoma cells.

## Results

We studied the transcriptomic and proteomic response to an altered Aβ_42_/Aβ_40 _ratio in human neuroblastoma cells. An increased or decreased Aβ_42_/Aβ_40 _ratio revealed differentially expressed transcripts, of which the 60 most up-regulated were used here. For the corresponding proteomic approach the 20 most up-regulated proteins were selected to validate altered protein expression. Only the overlap of transcriptomic and proteomic data was used for further analysis. To analyze altered Aβ generation in a controlled manner, C99-overexpression constructs encoding the C-terminal part of APP (C99) were used [6,7]. This peptide is identical to the APP-derived C99, the ultimate precursor for Aβ generation. C99 is processed by γ-secretase in the same manner as APP-derived C99, making it an ideal substrate to study γ-secretase function or its cleavage products Aβ_42 _and Aβ_40 _without the influence of β-secretase. Since, due to a point mutation, the constructs generated peptides only differing in a single amino acid outside the Aβ domain (at position 45 or 50, C99I45F and C99V50F, respectively) compared with the wild-type construct (C99WT), they were ideal for gene expression profiling, enabling us to minimize potential technical variation influencing gene expression.

### Single independent clones of the human neuroblastoma cell line SH-SY5Y, overexpressing C99, were selected and checked for Aβ_42 _and Aβ_40 _generation

**Figure 1:**
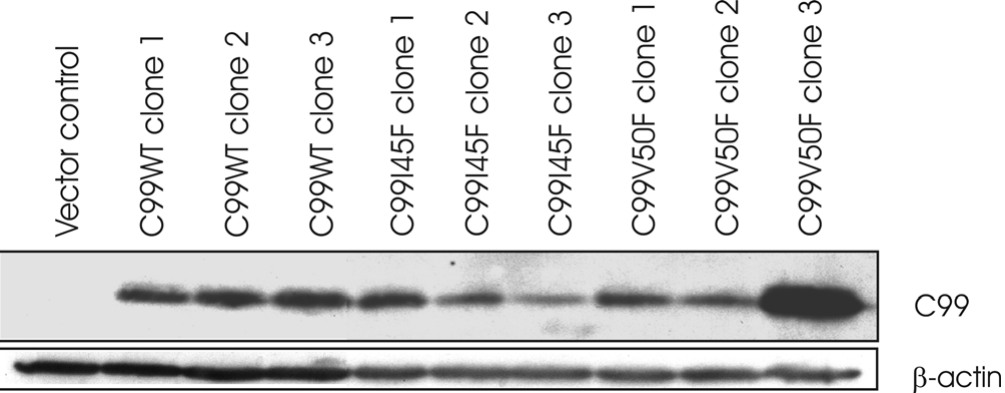
**C99 overexpression in independent cell clones**. SH-SY5Y cells were stably transfected with a pCEP-vector containing the amyloid precursor protein C-terminal fragment C99WT, and constructs bearing the point mutations C99I45F and C99V50F. The same cell line was transfected with an empty vector (negative control). Eight clones (clone 1–3 for C99WT, clone 1–3 for C99I45F and clone 1–2 for C99V50F) with approximately similar expression levels and C99V50F clone 3, showing stronger expression, were selected and used for transcriptome and proteome analysis. Apart from analyzing the complete set of three clones, data analysis for the transcriptomic approach was also performed by excluding clone 3 (C99V50F) resulting in no significant difference compared to the triplicates.

SH-SY5Y cells were stably transfected with constructs coding for the APP C-terminal fragment C99WT, and also with constructs bearing the point mutations C99I45F and C99V50F and the vector only (negative control) (Figure 1).

**Figure 2:**
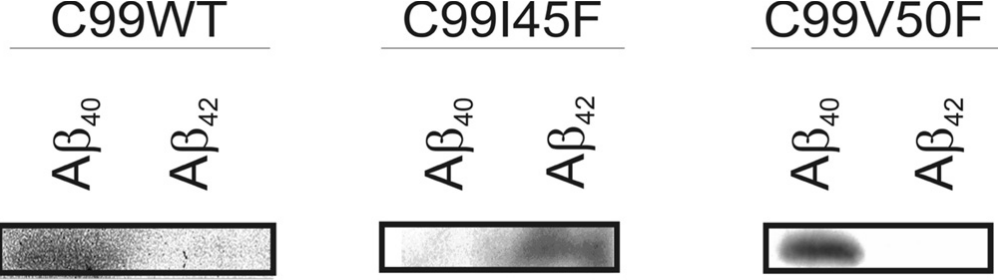
**Aβ_42 _and Aβ_40 _generated from their direct precursor C99 in independent cell clones**. Aβ_42 _and Aβ_40 _were immunoprecipitated from conditioned media of SH-SY5Y cells, overexpressing C99, using specific antibodies for Aβ_42 _and Aβ_40_. Both Aβ species were detected by Western blotting using antibody W02. C99 is intracellularly cleaved, generating different amounts of Aβ_42 _and Aβ_40 _in C99I45F and C99V50F. C99I45F generates more Aβ_42 _than Aβ_40_, whereas C99V50F generates more Aβ_40 _than Aβ_42_.

The purpose for using these mutations was their ability to strongly shift the Aβ_42_/Aβ_40 _ratio in either direction, as previously demonstrated in detail [6]. This was confirmed here (Figure 2).

As expected and described in detail [6,7] C99I45F and C99V50F had an opposite effect on the Aβ species generated: C99I45F is mainly processed to Aβ_42_, resulting in a dramatic increase of the secreted Aβ_42_/Aβ_40 _levels (relative ratio approximately 20.4 compared with the Aβ_42_/Aβ_40 _ratio in C99WT); C99V50F is mainly processed to Aβ_40 _(relative ratio approximately 0.3 compared with C99WT) [6].

### CRABP1 was up-regulated in the mutant with an increased Aβ_42_/Aβ_40 _ratio

**Figure 3:**
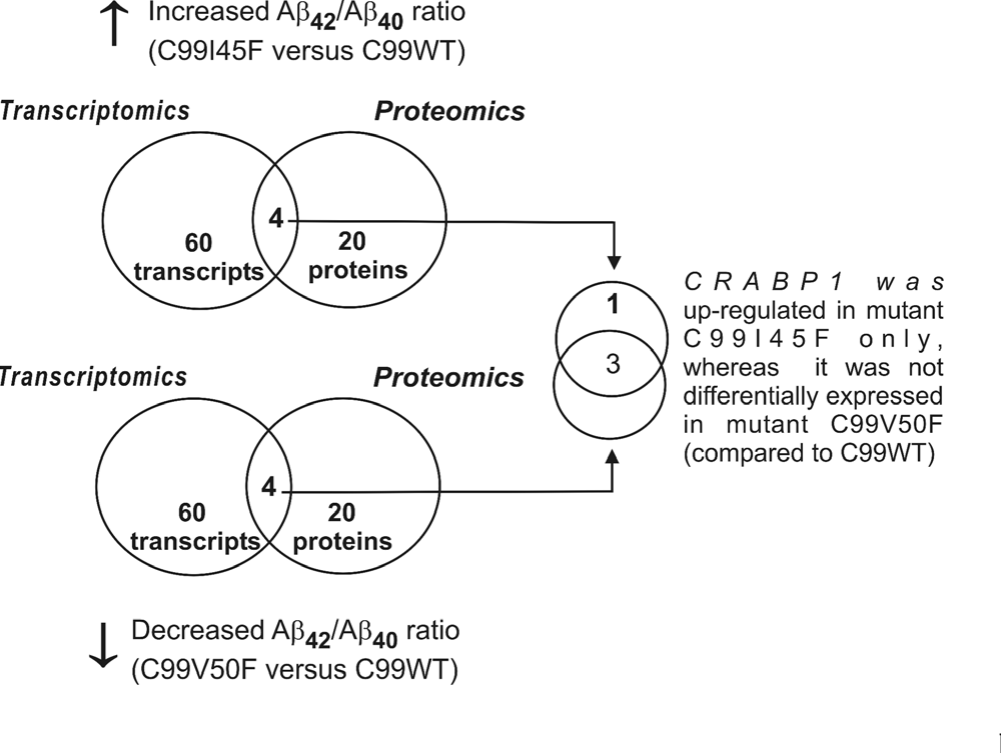
**Overlap of differentially expressed transcripts and proteins revealed CRABP1 up-regulation specific for an increased Aβ_42_/Aβ_40 _ratio**. Comparison of C99I45F or C99V50F versus C99WT revealed differentially expressed transcripts, of which each of the 60 most up-regulated were used here. The 20 most up-regulated proteins each were selected for the corresponding proteomic approach. An intersection of the transcriptomic and proteomic data was subsequently performed. Only the intersection of both approaches (four transcripts and proteins) was used for further analysis. Out of these four, only CRABP1 was up-regulated in C99I45F, whereas no differential expression was found in C99V50F (both mutants compared to C99WT). The remaining three transcripts and proteins were differentially expressed in both mutants. The proteomic approach was performed blinded by an independent laboratory. The term 'differentially expressed' was applied when the fold change exceeded a threshold of at least 1.9 either on the transcript or protein level.

Genome- and proteome-wide expression profiles of the human neuroblastoma cell line SH-SY5Y were combined and compared with each other (Figure 3).

Three single independent clones each from C99WT, C99I45F and C99V50F (Figure 1) were used for transcriptomic and proteomic analyses (mock-transfected cells as negative control).

For transcriptomics, whole genome HG-U133 A and B chips were used. Replicates were prepared and hybridized on different days and were derived from different independent clones. Data analysis was performed by calculating the mean of three independent single clones.

**Figure 4:**
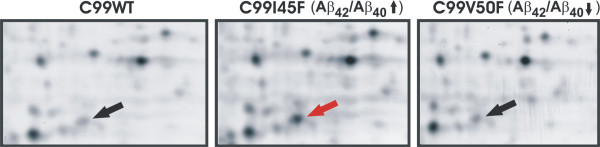
**CRABP1 was up-regulated specifically for an increased Aβ_42_/Aβ_40 _ratio demonstrated by 2D-DIGE**. Two-dimensional polyacrylamide gel electrophoresis of CyDye-labeled proteins, extracted from SH-SY5Y cells. C99I45F and C99V50F were compared with C99WT. Differentially expressed proteins, evaluated by intensity of merged colors (Cy5, Cy3), were identified by mass spectrometry. Arrows indicate CRABP1.

For proteomics, three clones each from C99WT, C99I45F and C99V50F were pooled, then proteins were extracted, CyDye labeled and analyzed by two-dimensional differential in-gel electrophoresis (2D-DIGE, Figure 4).

Up-regulated proteins were identified by mass spectrometry [24]. Only the intersection of the transcriptomic and proteomic approach was used for further analysis, thus increasing the reliability of the data (Table 1).

**Table 1:** CRABP1 was up-regulated in mutant C99I45F (Aβ_42_/Aβ_40_↑) only, whereas mutant C99V50F (Aβ_42_/Aβ_40_↓) showed no differential expression of CRABP1

Name	Fold change C99I45F/C99WT		Fold change C99V50F/C99WT		*P*-value	
	
	Transcriptomics	Proteomics	Transcriptomics	Proteomics	Transcriptomics	Proteomics
**CRABP1**	**2.7**	**2.6**	**1.3**	**-1.1**	**0.123**	**0.032**
NEF3	2.6	3.1	2.3	2.7	0.038	0.01
NEFL	2.2	2.3	2.2	2.5	0.032	0.004
INA	1.8	1.8	1.6	1.9	0.056	0.002

Comparisons of both mutants with C99WT. The overlay of transcriptomics and proteomics revealed four differentially expressed transcripts and proteins respectively. Out of these four, only CRABP1 was differentially expressed in C99I45F whereas C99V50F showed no differential expression of CRABP1 (compared with C99WT). One-way analysis of variance (ANOVA) was performed for C99WT, C99I45F and C99V50F. CRABP1, cellular retinoic acid binding protein 1 (NCBI accession of the protein identified by proteomics: gi|48146151); NEF3, neurofilament 3 (gi|67678152); NEFL, neurofilament light polypeptide 68 kDa (gi|105990539); INA, internexin neuronal intermediate filament protein alpha (gi|14249342).

CRABP1 was the second most up-regulated protein of the whole proteome and the second most up-regulated transcript of approximately 20,000 tested transcripts, when only chip A was considered (22,283 probe sets).

A direct comparison of both mutants revealed CRABP1 as strongly up-regulated in C99I45F compared with C99V50F. This comparison revealed an effect mediated by a changed Aβ_42_/Aβ_40 _ratio, because both mutants expressed inverse levels of Aβ_42 _and Aβ_40 _respectively (Table 2).

**Table 2:** Direct comparison between the two mutants (C99I45F versus C99V50F) showed CRABP1 as up-regulated in C99I45F (Aβ_42_/Aβ_40_↑) whereas neurofilaments were not differentially expressed

Name	Fold change C99I45F/C99V50F		*P*-value	
	
	Transcriptomics	Proteomics	Transcriptomics	Proteomics
**CRABP1**	**2.3**	**2.8**	**0.188**	**0.059**
NEF3	1.1	-1.1	0.790	0.56
NEFL	1.1	-1.1	0.640	0.51
INA	1.1	-1.1	0.679	0.24

Direct comparison of C99I45F and C99V50F (baseline experiment, C99V50F) revealed effects mediated by an altered Aβ_42_/Aβ_40 _ratio for CRABP1 by a fold change distinctly deviating from 1.0, and an effect mediated by C99 for neurofilament 3 (NEF3), neurofilament light polypeptide 68 kDa (NEFL) and internexin neuronal intermediate filament protein alpha (INA) by a fold change close to 1.0, because C99 was approximately equally expressed in both mutants. Significance was determined performing an unpaired *t*-test for the direct comparison of both mutants. As to be expected, *p*-values were high for not differentially expressed genes [62,63].

However, neurofilament 3 (NEF3), neurofilament light polypeptide 68 kDa (NEFL) and internexin neuronal intermediate filament protein alpha (INA) were not differentially expressed (Table 2). We regard this unaltered expression of neurofilaments as mediated by C99, since C99 is expressed in similar amounts in both mutants and hence a comparison between these two mutants results in a fold change close to 1.0 (not differentially expressed).

A comparison of SH-SY5Y cells transfected with the C99WT encoding construct versus SH-SY5Y cells transfected with the empty vector (mock) provides information about the effect mediated by C99 (Table 3).

**Table 3:** CRABP1 was not differentially expressed in consequence of C99-overexpression in contrast to neurofilaments

Name	Fold change C99WT/mock		*P*-value	
	
	Transcriptomics	Proteomics	Transcriptomics	Proteomics
**CRABP1**	**1.0**	**1.4**	**0.979**	**0.042**
NEF3	-3.4	-1.8	0.024	0.086
NEFL	-3.0	-1.3	0.039	0.11
INA	-1.9	-1.3	0.069	0.029

Comparison between C99WT and mock-transfected cells revealed effects mediated by C99. Neurofilament 3 (NEF3), neurofilament light polypeptide 68 kDa (NEFL) and internexin neuronal intermediate filament protein alpha (INA) were down-regulated as a consequence of C99-overexpression. CRABP1 was not differentially expressed (cut-off for differential expression at least 1.9 on the transcript or protein level, respectively).

Neurofilaments (NEF3, NEFL, INA) were down-regulated as a consequence of C99 overexpression. CRABP1 was not differentially expressed, supporting our view that C99 is not responsible for CRABP1 dysregulation.

### Differential expression of *CRABP1 *was confirmed by real-time PCR

Expression of *CRABP1 *was measured by quantitative real-time PCR with *cyclophilin A *as an endogenous normalization control (*cyclophilin A *was selected out of 10 normalization controls; see Additional file 1). Measurements reflect the mean of three independent clones, measured in triplicate. The fold change for *CRABP1 *of mutant C99I45F (Aβ_42_/Aβ_40_↑) compared with C99WT was 4.1 (standard deviation of the fold change: ± 2.3). In contrast to this, the differential expression of *CRABP1 *was below our defined cut-off (<1.9) for C99V50F (Aβ_42_/Aβ_40_↓) compared with C99WT and thus was regarded as not differentially expressed.

### Cells with an increased Aβ_42_/Aβ_40_ ratio up-regulated CRABP1, which made cells less sensitive to RA

**Figure 5:**
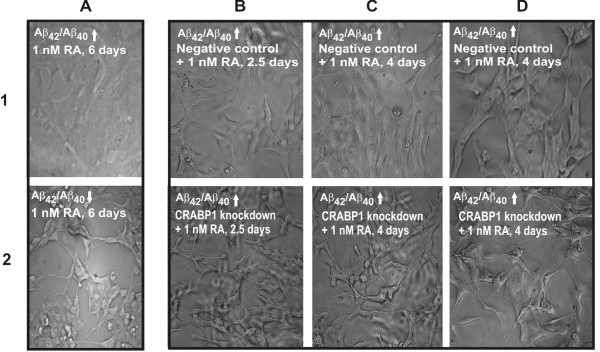
**Increased Aβ_42_/Aβ_40 _ratio reduced responsiveness of SH-SY5Y cells to RA and the knock down of up-regulated CRABP1 rescued their differentiation potential**. Phase contrast images showing living human neuroblastoma cells (SH-SY5Y), grown on collagen coated glass cover slips and treated with 1 nM retinoic acid (RA). Differentiation was evaluated by the number, shape and length of outgrowing protrusions: (1A) C99I45F (Aβ_42_/Aβ_40_↑); (2A) C99V50F (Aβ_42_/Aβ_40_↓). Differentiation was evaluated after RA-treatment for 6 days. Both cultures were 50% confluent when RA was added (day zero). C99I45F reached 90–100% confluency after 4–6 days without any signs of differentiation, whereas C99V50F did not exceed more than 60–70% confluency (after 6–10 days) but showed strong differentiation. C99I45F was also evaluated at 60–70% of confluency showing no signs of differentiation (data not shown), thus strong confluency of C99I45F (shown here) does not conceal putative signs of differentiation. (B) C99I45F (Aβ_42_/Aβ_40_↑): 30 nM siRNA was administered to the cells for 24 hours in combination with a treatment of 1 nM RA for 2.5 days. After 2.5 days, the effects of more than 50% knockdown of CRABP1 (2B) was compared with a nonsense sequence (negative control, (1B)). (C) C99I45F: same conditions as in (B) except that RA was administered for 4 days. Differentiation was evaluated after 4 days. Knockdown of CRABP1 (2C) was compared with a nonsense sequence (negative control, (1C)). (D) C99I45F: same conditions as in (C), but with another preparation from the same experiment as in (C). (B) and (C) show preparations from different experiments. Experiments were repeated three times with consistent results.

CRABP1 is involved in RA metabolism and transport [27] and we found it up-regulated as a consequence of an increased Aβ_42_/Aβ_40 _ratio. This raised the question of whether cells with an increased Aβ_42_/Aβ_40 _ratio show altered responses to RA treatment. SH-SY5Y cells were stably transfected with the constructs increasing or lowering the Aβ_42_/Aβ_40 _ratio (Figure 2). These cells were treated with 0.1–1000 nM RA in the absence or presence of serum. After 6 days, differentiation was evaluated by observing the length and number of outgrowing protrusions by phase contrast microscopy (Figure 5, 1A and 5, 1B).

Furthermore, the cell shape and number of cells were evaluated. We selected 1 nM RA for the subsequent functional validation and C99I45F-transfected cells were treated with 1 nM RA for 6 days (Figure 5, 1A). No signs of differentiation were observed, irrespective of the cell confluency and duration of RA treatment (cells were checked daily by light microscopy for up to 10 days). In contrast to this, the cells expressing C99V50F (Figure 5, 2A) showed differentiation at 1 nM RA treatment for 6 days: the cells were approximately 30–60% confluent and did not reach 100% confluency after 10 days. Cells had an average of two to four protrusions. This differentiation was observed from 0.1–10 nM RA, which approximately corresponds to physiological plasma concentrations [28,29]. At concentrations of 100 nM or more RA, differentiation could also be observed for the C99I45F transfected cell line.

### CRABP1 knockdown rescued the differentiation potential of Aβ_42_ overproducing human neuroblastoma cells after RA treatment

If an increased Aβ_42_/Aβ_40 _exerts the diminished differentiation behavior via CRABP1, a CRABP1 knockdown in C99I45F cells should rescue this effect. We administered 30 nM siRNA to C99I45F SH-SY5Y cells for 24 hours in combination with a treatment of 0.1–1000 nM (1 nM shown in Figure 5) for 2.5 to 4 days in the absence (data not shown) or presence of serum. Serum withdrawal can mimic differentiation ('pseudo differentiation') and was therefore excluded from further analysis. A more than 50% knockdown of CRABP1 was detected by quantitative real-time PCR (*p *= 0.0002, *n *= 3).

Differentiation was evaluated after 2.5 days and 4 days. Knockdown of CRABP1 in combination with 1 nM RA (Fig. 5, 2B–2D) resulted in a strong change of cell shape, whereas transfection with a nonsense sequence, combined with 1 nM RA (negative control, Figure 5, 1B–1D) did not alter the shape of the cells. The strongest differentiation was observed at 1 nM RA. No differentiation could be observed for treatment with siRNA, but without RA or treatment with 1 nM RA, but without siRNA (data not shown). After CRABP1 knockdown and RA-treatment the cells were approximately 30–80% confluent (Figure 5, 2B–2D) and did not reach 100% confluency after 10 days. The extent of interconnections between cells was clearly increased (Figure 5, 2D) compared with the negative control (Figure 5, 1D). See Additional file 1 for an enlarged picture.

### Three further genes associated with RA metabolism were differentially expressed as a consequence of a changed Aβ_42_/Aβ_40_ ratio and may have influenced the effects mediated by RA

Three further genes may have influenced the effects mediated by RA. Chip analysis revealed the following differential expression: Cytochrome P450 family 26 subfamily B polypeptide 1 (*CYP26B1*), a RA-metabolizing enzyme [27], was found to be up-regulated 1.8-fold (*p *= 0.01, *n *= 3) in C99I45F (Aβ_42_/Aβ_40_↑), whereas C99V50F (Aβ_42_/Aβ_40_↓) showed no differential expression (compared with C99WT). Direct comparison of both mutants (C99I45F/C99V50F) revealed a 2.6-fold up-regulation for *CYP26B1 *in mutant C99I45F (*p *= 0.02, *n *= 3). Retinoic acid receptor (RAR)-related orphan receptor B (*RORB*) was down-regulated 2.0-fold (*p *= 0.049, *n *= 3) in C99V50F (compared with C99WT), whereas it was not differentially expressed in C99I45F. RAR beta (*RARB*) was not differentially regulated in C99V50F, whereas it was up-regulated 1.4-fold (*p *= 0.05, *n *= 3) in C99I45F (compared with C99WT).

## Discussion

Human instead of murine cells were used for transcriptomics and proteomics to facilitate potential comparability with patient-derived data sets. The human neuroblastoma cell line SH-SY5Y has characteristics close to primary neurons, is used to demonstrate differentiation processes [30-33] and is a frequently used neural cell line for microarray studies [34-37].

### Association between AD and RA

Associations between AD and RA transport and metabolism are known [38,39]. It was shown that disruption of the retinoid signaling pathway causes a deposition of Aβ in the adult rat brain [40]. RA amounts are determined by many regulatory proteins, such as retinoid binding proteins, retinoid anabolizing and catabolizing enzymes [41]. CYP26B1 has been linked to AD and psychosis [42]. One crucial mechanism whereby the availability of RA is regulated is by binding to CRABP1. CRABP1 is a protein with a molecular weight of 15.4 kDa, localized in the cytoplasm. The gene is strongly conserved in evolution and is assumed to play an important role in RA-mediated differentiation and proliferation processes. It may regulate the access of RA to the nuclear RARs. In the adult brain the two main regions of RA signaling are the olfactory bulb and the hippocampus [43]; both regions are predominantly affected in late onset Alzheimer's disease (LOAD) [41]. CRABP1 and RA are inversely regulated [44]. CRABP1 binds RA and prevents its entering the nucleus and in cells with low CRABP1 expression RA enters the nucleus and binds to RARs [8-10].

An association between CRABP1 and Aβ has not yet been established. In this study we have demonstrated that an increased Aβ_42_/Aβ_40 _ratio resulted in CRABP1 up-regulation. Furthermore, we demonstrated that up-regulated CRABP1 reduced the differentiation potential of SH-SY5Y cells. C99I45F-transfection of SH-SY5Y cells resulted in differentiation only if exposed to 100 nM or more of RA, but the same cell line showed already strong differentiation at 1 nM RA when CRABP1 was knocked down by more than 50%. Therefore, we estimate that a 50% knockdown of CRABP1 makes cells more sensitive to RA by approximately a factor of 10^1^-10^2^. The physiological plasma concentration of RA in humans is approximately 10 nM and 8.4 pmol g^-1 ^in the hippocampi of mice [45]. Excess of exogenous RA may over-saturate the binding capacities of CRABP1 allowing the remaining RA to bind to the RARs [46]. This provides an explanation for our finding that treatment with an excess of RA (>100 nM) makes no difference in the differentiation behavior detectable, but differences are evident at low (physiological) levels of RA. *CRABP1 *transfection of AMC-HN-7 cells results in an increased CYP26-mediated catabolism of RA [27]. This decreases the RA level accessible to the nuclear receptors. Indeed, we found *CYP26B1 *to be up-regulated in C99I45F, but not in C99V50F. *RORB *was down-regulated in C99V50F, but not in C99I45F. Furthermore *RARB *was not differentially regulated in C99V50F, but up-regulated in C99I45F. These observations might reflect a response of the cells to an increased RA level in C99V50F or a decreased RA level in C99I45F, respectively. An inverse regulation of receptors and their ligands is often observed [47].

### Linkage of the chromosomal locus 15q24 to mental retardation

*CRABP1 *is located on the same chromosomal locus (15q24) as alpha polypeptide 3, 4 and 5 of the nicotinic cholinergic receptor (*nAChR*) and cytochrome P450, family 11, subfamily A, polypeptide 1 (cholesterol side chain cleavage, *CYP11A1*). Association of *nAChR *and AD has been described previously [48]. Moreover, there has been found to be a linkage of the chromosomal locus 15q24 to mental retardation [49] and linkage of the flanking regions (15q22 and 15q26) to AD [50,51]. This linkage may be explained by the presence of alpha polypeptide 3, 4 and 5 of the *nAChR*, or of *CRABP1*, located on the same chromosomal locus.

### Neurofilaments were inversely regulated by C99 and Aβ_42_, Aβ_40_

We observed down-regulation of the neurofilaments NEF3, NEFL and INA as a result of C99 overexpression. Interestingly, these three neurofilaments were up-regulated in response to Aβ_42 _and Aβ_40 _overproduction. This may indicate a role of NEF3, NEFL and INA in the axonal 'clogging' phenomenon [52-55] observed in neurons induced by APP or its cleavage products [56].

### Sensitive balance between proliferation and differentiation was influenced by an altered Aβ_42_/Aβ_40 _ratio via CRABP1

Treating neural stem cells with Aβ increases the total number of neurons in a dose-dependent manner [57]. In our study we used neuroblastoma cells, which share related proliferation and differentiation properties with neural stem cells. We observed increased proliferation of human neuroblastoma cells in consequence of an increased Aβ_42_/Aβ_40 _ratio via CRABP1 and suggest that this influences neurogenesis by promoting proliferation. However, the newly generated neurons may be prevented from adopting a functional phenotype, as a consequence of CRABP1 up-regulation restricting the quantity of RA. This view is supported by a study showing that RA induces neurite outgrowth in SH-SY5Y cells [58]. Theoretically, it seems possible that CRABP1 knock-down would release the block of terminal differentiation of neurons in AD and thus improve the differentiation of neural stem cells into a functional phenotype. RA has often been used to terminally differentiate neuroblastoma cells [59,60] as well as primary neuroblasts [61]. We observed outgrowing protrusions typical for RA-induced differentiation. Moreover, we observed that growth stopped or slowed down in a RA concentration depending manner, which is characteristic of an effective differentiation process.

Our study focused on an increased Aβ_42_/Aβ_40 _ratio, which is typical for AD. It does not allow us distinguish between pure Aβ_42 _and pure Aβ_40 _effects, because intracellular processing by γ-secretase typically generates less Aβ_40 _when more Aβ_42 _is generated and vice versa. However, we emphasize that our approach better resembles *in vivo *conditions than approaches in which Aβ_42 _or Aβ_40 _is added from outside the cells. In our approach C99 is intracellularly cleaved resulting in different Aβ_42_/Aβ_40 _levels, which are released into the extracellular space. This is closer to *in vivo *conditions than treating cells artificially with Aβ_42 _or Aβ_40_.

The generation of different Aβ_42_/Aβ_40 _ratios is inherently accompanied by the generation of other C99 cleavage products like the p3 peptides, the APP intracellular domains (AICDs) and further cleavage products. The C99 point mutations are expected to equally shift the p3_40_/p3_42 _and Aβ_42_/Aβ_40 _ratios, but little is known about how these mutations affect the AICD production. Neurons produce very little p3 from C99, and the AICD sequence starts at the ε- and not at the γ-site of APP, therefore it would be expected that the main effect of the mutations analyzed is due to altered Aβ generation. This, however, does not exclude the possibility that several C99 cleavage products work in concert with each other.

In summary, we found that an increased Aβ_42_/Aβ_40 _ratio up-regulated CRABP1, reducing the availability of free RA. This resulted in an increased tendency towards proliferation accompanied by a reduced potential to differentiate. This effect could be rescued by knocking down CRABP1. We speculate that Aβ_42 _induces the initial steps in neurogenesis by boosting neuronal precursor cell proliferation while preventing the terminal differentiation into mature neurons. This scenario may provide an explanation for why in AD there is an increase in neurogenesis and at the same time an increased risk for neurodegeneration.

## Conclusion

We conclude that the differentiation potential of the human neuroblastoma cell line SH-SY5Y is reduced via CRABP1 up-regulation as a consequence of an increased Aβ_42_/Aβ_40 _ratio.

## Abbreviations

2D-DIGE: two-dimensional differential in-gel electrophoresis; Aβ: β-amyloid peptides; AD: Alzheimer's disease; AICD: APP intracellular domain; ANOVA: analysis of variance; APP: amyloid precursor protein; C99: C-terminal fragment of APP; CRABP1: cellular retinoic acid binding protein 1; FBS: fetal bovine serum; INA: internexin neuronal intermediate filament protein alpha; MALDI-TOF-MS: matrix assisted laser desorption ionization time-of-flight mass spectrometry; MEM: Minimum Essential Medium; NEF3: neurofilament 3; NEFL: neurofilament: light polypeptide 68 kDa; real-time PCR: real-time polymerase chain reaction; PMF: peptide mass fingerprint; RA: all-trans retinoic acid; RAR: retinoic acid receptor; RARB: RAR beta; RORB: RAR-related orphan receptor B; SDS-PAGE: sodium dodecyl sulfate polyacrylamide gel electrophoresis.

## Competing interests

The authors declare that they have no competing interests.

## Authors' contributions

MU designed the project, wrote the paper, performed gene expression profiling, real-time PCR, differentiation assays and analyzed the data. PB performed 2D-DIGE and analyzed the data. OJ performed mass spectrometry and analyzed the data. MO was involved with the proteomics project design. YK contributed to data analysis. AW made a technical contribution to the microarray analysis. LB performed real-time PCR. KH validated the protein expression. TH designed and supervised the project, and edited the manuscript.

## Pre-publication history

The pre-publication history for this paper can be accessed here:



## Supplementary Material

Additional file 1 **Supplemental data 1**. 1. Larger scale of Figure 5D; 2. Transcriptomic data analysis;. 3. Remarks; 4. Materials and methods; 5. Quality control of cells, target-RNA and arrays; 6. RNA-quality assessed by using the Agilent 2100 Bioanalyzer. Click here for file

Additional file 2 **Table 4**. Data. Click here for file

Additional file 3 **Table 5**. Data. Click here for file
