# Supplementary material for: Upregulation of CRABP1 in human neuroblastoma cells overproducing the Alzheimer-typical Aβ42 reduces their differentiation potential
Source: BMC Med. 2008 Dec 16;6:38. doi: 10.1186/1741-7015-6-38 (PMC2645429; doi:10.1186/1741-7015-6-38)
Supplement: Additional file 1 — Supplemental data 1. 1. Larger scale of Figure 5D; 2. Transcriptomic data analysis;. 3. Remarks; 4. Materials and methods; 5. Quality control of cells, target-RNA and arrays; 6. RNA-quality assessed by using the Agilent 2100 Bioanalyzer. [file 1741-7015-6-38-S1.doc]

**1. Larger scale of Figure 5 D**

***
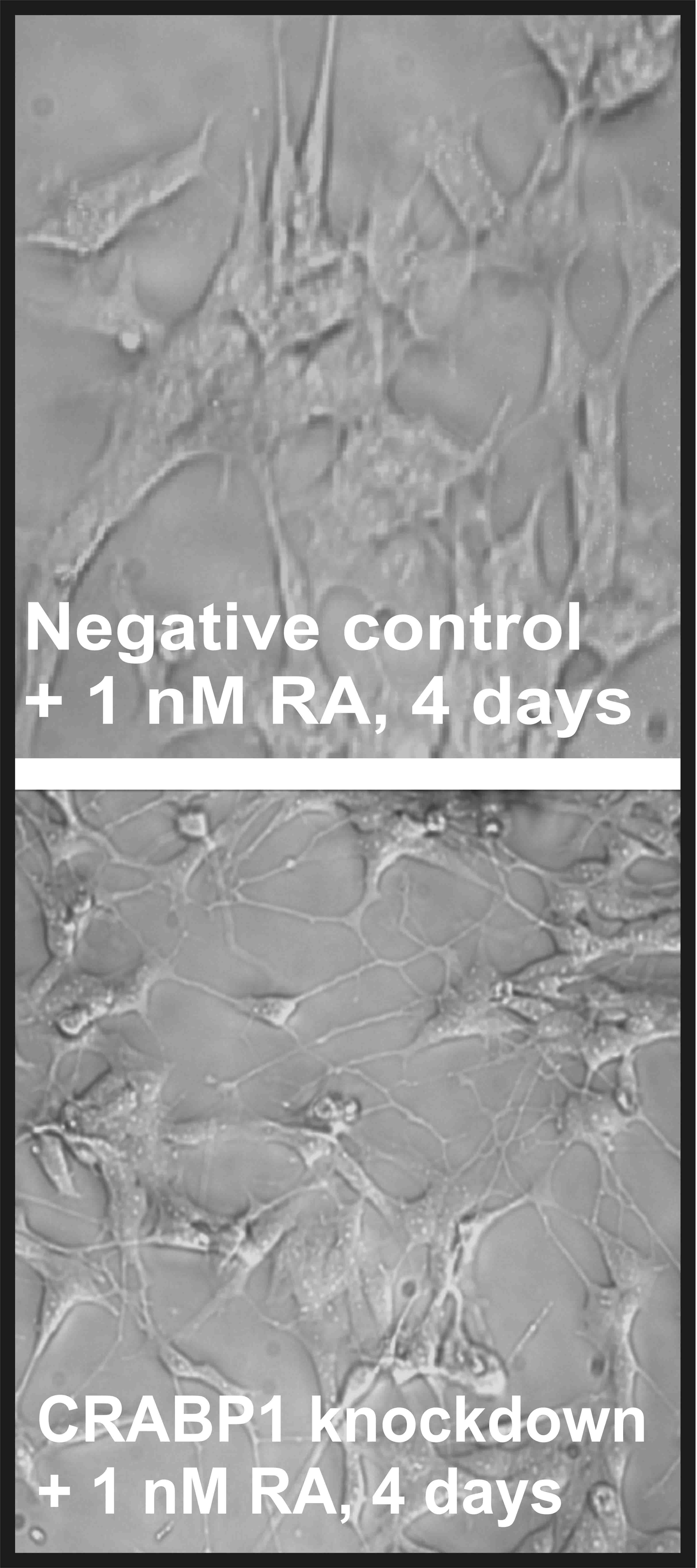
***

**2. Transcriptomic data analysis**

Transcriptomic data were analyzed with MAS 5.0, GCOS 1.2 (both Affymetrix) and Array Assist 3.3 (Stratagene). Cel-files were integrated into Array Assist and chp-files were created by using the PLIER algorithm. P-values were calculated from 3 independent experiments using either a two class unpaired t-test or analysis of variance (One Way ANOVA). Further data analysis was performed with Excel (Microsoft) (Table 4 and 5).

**3. Remarks**

***Overlapping results obtained from different laboratories and different technologies increased reliability.*** Our results were derived from several independent clones with slightly varying expression. So clonal effects, which can only be observed in a single clone, but not in others can be regarded as minimized. Furthermore, two independent experimental approaches, transcriptomics and proteomics, led to highly similar results (CRABP1 was found to be the second most up-regulated transcript of approximately 20,000 ones tested on Chip A, and the second most up-regulated protein of the whole proteome)**.** Transcriptomics and proteomics were carried out by two independent laboratories and the time interval between these two approaches was nearly three years, minimizing the probability that cell culture conditions or preparation procedures caused a bias in our data. The genomic approach was performed first and the subsequent proteomic approach was performed blind.

**4.** **Materials and Methods**

***Cell line.*** The human neuroblastoma cell line SH-SY5Y was originally isolated from transformed neural tissue (neuroblastoma), from a 4 year old girl. SH-SY5Y is a thrice cloned (SK-N-SH -> SH-SY -> SH-SY5 -> SH-SY5Y) subline of the neuroblastoma cell line SK‑N‑SH, the latter having been isolated and established in 1970. This cell line has 47 chromosomes.

***Plasmids.*** C99 encoding sequences (spA4ct-DA) were cloned into a pCEP4 vector (Invitrogen), resulting in the following constructs: pCEP4-spA4ct-DA-WT, pCEP4-spA4ct-DA-I45F and pCEP4-spA4ct-DA-V50F. The plasmid constructs have been described previously.

***Cell culture and transfections.*** SH-SY5Y cells were cultured in 50% Minimum Essential Medium (MEM, Sigma) and 50% Nutrient Mixture F-12, HAM) (Sigma). This mixture was supplemented with 10% FBS (PAN), 1% nonessential amino acid solution (Sigma) and 1% L‑Glutamin (Sigma), in a humidified atmosphere with 5% CO_2_. 70% confluent cells were transfected with the following constructs: pCEP4 empty vector (Invitrogen, negative control), pCEP4-spA4ct-DA-WT, pCEP4-spA4ct-DA-I45F and pCEP4-spA4ct-DA-V50F. Lipofectamine in combination with Plus-reagent (both Invitrogen) were used for transfection according to the manufacturer’s recommendations. Stably transfected cell clones were selected using 300 µg/ml hygromycin B (PAA Laboratories). Single clones were checked for expression of C99. Clones with approximately similar expression levels were selected for further analysis. Subsequently, generation of Aβ_42_ and Aβ_40_ was determined.

***Preparation of cell lysates and collection of conditioned media.*** 5 ml culture medium was added to a 70% confluent monolayer of cells in a 10 cm culture dish and conditioned media were collected after 16-48 h. The conditioned media were centrifuged at 4 °C for 1 min at 13,000 rpm and the supernatants were used for immunoprecipitation of soluble secreted Aβ. Cell lysates were prepared in parallel to the conditioned media. Cells were harvested and lysed on ice in lysis buffer (50 mM Tris/HCl, ph 7.5, 150 mM NaCl, 1 % Nonidet P-40, 1% Triton X-100 and 5 mM EDTA) supplemented with Complete® protease inhibitor (Roche).

***Immunoprecipitation.*** Conditioned media were immunoprecipitated with 20 µl of protein G‑Sepharose (Sigma) and the antibodies G2-10 and G2-11. The immunoprecipitated proteins were separated on 12% Tris-Tricine gels. Western blot analysis was performed with the antibody W02.

***Western-Blotting and antibodies.*** Western blot analysis was performed as described elsewhere. Briefly, heat-denatured samples were separated by 10% Tris-Tricine SDS-PAGE. Proteins were blotted onto nitrocellulose membrane and detected with the monoclonal antibody W02 specific for residues 1-10 of Aβ. The monoclonal antibodies G2-10 specific for Aβ_40_ and G2-11 specific for Aβ_42_ were used for immunoprecipitation. For normalisation of loaded protein amounts, blotted membranes were stripped as described elsewhere (www.abcam.com) and re-probed with an anti-β-actin antibody (Abcam), diluted 1:1000.

***Whole genome oligonucleotide microarray analysis (Gene Chips****®****, Affymetrix).*** Gene Chip analysis was performed according to the instructions of the Expression Analysis Technical Manual (Affymetrix) with minor modifications:

*Total RNA preparation from cultured cells.* Total RNA was extracted using the Qiashredder-Kit, RNeasy Midi-columns and the RNase-free DNase set (all from Qiagen). Human neuroblastoma cells (SH-SY5Y) were grown in one to six 10 cm cell culture dishes until they reached 70% confluency. The cells were then lysed according to the manufacturer’s recommendations and total-RNA was extracted using RNeasy Midi-columns. Any remaining DNA was digested directly on the columns by using the RNase-free DNase set. Total-RNA was eluted with 250-500 µl RNase-free water. 20 µg total-RNA was precipitated by adding 10% (v/v) sodium-acetate (3 M, pH 5.2) and 2.5 volumes of absolute ethanol. The remaining precipitate was air-dried and dissolved in 9 µl of RNase-free water.

*Reverse transcription of total RNA into cDNA by oligo(dT) primers.* Total RNA was used to generate first-strand cDNA by using the Superscript^TM^ Double-Stranded cDNA Synthesis Kit (Invitrogen). Briefly, 20 µg of total RNA was used to generate first-strand cDNA by using a T7-linked oligo(dT)_24_ primer: 5’-GGCCAGTGAATTGTAATACGACTCACTATAGGGAGGCGG(dT)_24_ -3’ (Proligo). After second-strand synthesis cDNA was purified using the GeneChip® Sample Clean up Module (Affymetrix) by following the manufacturer’s instructions.

*In vitro transcription of cDNA into cRNA.* Purified cDNA was in vitro transcribed using the BioArray^TM^ *High Yield*^TM^ RNA Labeling Kit (Enzo Life Sciences). 3.3 µl of purified cDNA was mixed with appropriate volumes of reaction buffer, biotin-labeled ribonucleotides, DTT, RNase inhibitor and T7 RNA polymerase in a total volume of 40 µl according to the manufacturer’s instructions. This mixture was incubated for 5 h at 37°C and purified using the GeneChip® Sample Clean up Module (Affymetrix) by following the manufacturer’s instructions.

*Fragmentation of cRNA.* Fragmentation of cRNA was performed using the GeneChip® Eukaryotic Hybridization Control Kit (Affymetrix). 15 µg of purified cRNA was mixed with 8 µl 5x fragmentation buffer and filled up with RNase-free water to a total volume of 40 µl. This mixture was incubated for 35 min at 94°C. To confirm successful fragmentation cRNA was checked with the Bioanalyzer 2100™.

*Hybridisation of fragmented cRNA onto the arrays.* A hybridisation cocktail, containing 15 µg fragmented cRNA and hybridisation controls (spike controls), was prepared according to the Expression Analysis Technical Manual (Affymetrix). This cocktail was first hybridised to “Test 3 arrays” (Affymetrix) to recheck sample quality and subsequently hybridised to whole genome HG-U133 A (22,283 probe sets) and HG-U133 B (22,645 probe sets) oligonucleotide arrays. The Chips were incubated for 16 h at 45°C, rotating at 60 rpm, in a Gene Chip® Hybridization Oven 640 (Affymetrix).

*Washing, staining and scanning of the arrays.* The Chips were washed in a Gene Chip® Fluidics Station 400 (Affymetrix) stained with streptavidin-phycoerythrin, according to the manufacturer’s instructions. The arrays were scanned twice with a Gene Array® Scanner (Hewlett Packard) and the quality of the created dat-file images were evaluated by using the Microarray Suite MAS 5.0 (Affymetrix) and the Gene Operating Software GCOS 1.2 (Affymetrix).

*Quality control.* Each sample was screened and found to be free from mycoplasma contamination by using the Mycoplasma Detection Kit (Roche). RNA purity was assessed by measuring the 260 nm/280 nm ratio with a spectrophotometer (Biorad Smart Spec 3000). A 260 nm/280 nm ratio of 1.9-2.1 for Chip experiments and 1.8-2.1 for quantitative real-time PCR was accepted. Total-RNA, unfragmented cRNA and fragmented cRNA were checked with a Bioanalyzer 2100™ using the RNA 6000 Nano Assay Reagent Kit (both Agilent). For total-RNA two distinct bands (28s and 18s ribosomal RNA) were expected. The 28 s band should be approximately twice as strong as the 18 s band. For unfragmented cRNA a certain distribution of bands, representing the different mRNAs, was expected with an accumulation in the centre of the distributed bands. For fragmented cRNA, bands corresponding to a size of 35-200 bases were expected. After scanning, array images were assessed by several parameters giving information about Chip quality, hybridisation efficiency and RNA quality: Array images were assessed by eye to confirm the absence of bubbles or scratches. Furthermore scaling factors, background, noise, number of present transcripts, 3'/5' ratios for GAPDH and beta-actin and bio spike controls were used to assess quality.

***Quantitative real-time PCR and selection of an endogenous control for normalisation.*** Quantitative real-time PCR was performed in two steps. In the first step, total-RNA, extracted from human neuroblastoma cells, was reverse transcribed into single-stranded cDNA using random hexamer primers included in the High-Capacity cDNA Archive Kit (Applied Biosystems) following the manufacturer’s instructions. In the second step, this cDNA was amplified and measured in real time with a GeneAmp® 5700 Sequence Detection System (Applied Biosystems). Pre-designed Assays-on-Demand^TM^ (Applied Biosystems) were used for cDNA amplification and detection at real-time. The quantitative real-time PCR assay was performed using TaqMan® Gene expression Assays and theTaqMan® Universal PCR Master Mix. The reaction was carried out in 96 well plates (Applied Biosystems). Total reaction volume was 25 µl/well. 10-100 ng cDNA was used as template. Cycling conditions were: 50°C for 2 min, 95 °C for 10 min, followed by 40 cycles of 95°C for 15 s and 60°C for 1 min.

Relative quantification was performed with the 2^-∆∆^C_T_ method on an ABI PRISM® 5700 Sequence Detection System. For the ∆∆C_T_ calculation to be valid, the amplification efficiencies of the gene of interest and endogenous control must be approximately equal. Therefore standard curves were established for calculating the efficiency of amplification. An endogenous control for normalisation was selected out of 10 candidate controls (acidic ribosomal protein, β-actin, cyclophilin A, glyceraldehyde-3-phosphate dehydrogenase, phosphoglycerokinase, β_2_-microglobulin, β-glucoronidase, hypoxanthine ribosyl transferase, transcription factor IID TATA binding protein, transferrin receptor) using the TaqMan® Human Endogenous Control Plate (Applied Biosystems; the18s rRNA-assay was excluded due to a different preparation procedure and for practical reasons). The assay was performed according to the manufacturer’s instructions and measured with a GeneAmp 5700 Sequence Detection System (Applied Biosystems). All clones were tested for equal expression of the previously mentioned genes. CyclophilinA, β-actin and acidic ribosomal protein were preselected due to equal expression in all measured clones and thus were suitable for normalisation. After further detailed analysis cyclophilin A was finally selected for further measurements (cyclophilinA was only insignificantly more favorable than the other two).

***Proteomics:***

***Cy-Dye Labeling.*** For CyDye-labeling equimolar amounts of 3 independent single cell clones (Fig. 1) were pooled and the carefully washed cells were precipitated in 4x volume ice cold acetone overnight at -20°C. After centrifugation the pellet was air-dried for 1 h and resuspended in 7 M Urea, 2 M Thiourea, 4% CHAPS, 30mM Tris-HCl pH 8.1 at 8 °C and sonicated with a micro-tip (Branson Sonifier, USA) for 2x 10 cycles at 60% duty yielding a solution of 5 mg protein/ml. Insoluble fractions were removed by centrifugation. Labeling was carried out as follows: Each sample (approx. 100 µg) was labeled with 200 pmol of the respective Cy Dye. To avoid potential dye-specific preferences, the samples were labeled with both Cy3 and Cy5 according to the experimental design. For the mixed internal standard, aliquots of each individual sample were pooled and labeled with Cy2 in the same dye-to-Protein ratio. After 30 min of incubation at 8 °C in the dark the labeling reaction was abrogated by adding 20 nmol Lysine and incubated for further 10 min. To yield a higher protein load on the IPG-strip for gels intended for subsequent mass spectroscopy, the Cy-labeled and pooled samples were spiked with additional protein already lysed in the buffer as described above to yield a final load of 500µg Protein. The labeled samples were combined and diluted 1,33x by a stock solution containing 7 M Urea, 2 M Thiourea, 4 % CHAPS, 4 % IPG-Buffer 3-10 NL, 4 % DTT w/v.

***2D Gel Electrophoresis and Imaging.*** Isoelectric focussing was performed on immobilized pH 3-10 non-linear gradient gels (24 cm Immobiline dry strips pH 3-10 NL, GE Healthcare). The CyDye-labeled samples with a final volume of 113 µl each were incubated for 45 min at ambient temperature, centrifuged for 5 min at 10.000 x g and cup-loaded anodically onto the IPG-Strips rehydrated for 24 h in 7 M Urea, 2 M Thiourea, 4% CHAPS , 1% DTT, 1% IPG Buffer (40%) pH 3-10 NL, 0.002% BPB. Isoelectric focussing was carried out for a total of approx. 55,000 Vh (1h 150 V, ramp for 3h to 300V, ramp for 6h to 1000V, ramp for 3h to 8000V, hold at 8000V for 4:40h). After focussing the strips were equilibrated in two steps for 25 min each in 6 M Urea, 125 mM Tris-HCL pH 7.85, 3% SDS and 30% Glycerol (v/v); additionally 1% Dithiothreitol (DTT) for the first step (reduction) and 3.5% Iodoacetic Acid (IAA) for the second equilibration (alkylation) step were added. Second dimension SDS-PAGE was performed with isocratic12.5% gels (254 x 200 mm) according to Laemmli *et al.* at 3.5 W/gel overnight at 25 °C. The fluorescence signals of the three differently Cy-labeled protein samples were imaged using a laser scanner recording band pass filtered emission wavelengths of 520 nm (Cy2); 580 nm (Cy3) and 670 nm (Cy5) respectively (Typhoon 9400 Amersham Lifescience). For subsequent mass spectrometry the proteins were stained with colloidal Coomassie Brillant Blue according to Neuhoff *et al.* by immersing the gels in stainless steel trays and spots were excised manually.

***Differential in-gel Analysis.*** Using the DeCyder™ 3.0 Differential Analysis Software (Amersham Biosciences) the protein abundance changes within the different proteomes were compared pairwise for each gel with the “Differential in-gel Analysis” Software Module. This module normalizes CyDye signals, co-detects corresponding spots in each channel and calculates the spot-volume ratios of each spot. For analysis of the significance levels of protein abundance changes (expressed in x-fold) were determined by exceeding value for 2 standard deviations (95 th percentile confidence) of the mean volume ratio of all spots detected. In the “Biological Variance Analysis Module” quantified spots of all gels in the experiment were matched to a chosen master-gel. Significance was tested using either a two class unpaired t-test or analysis of variance (One Way ANOVA).

***Protein identification******.*** Excised gel plugs were subjected to an automated platform for the identification of gel-separated proteins as recently described in detail. Briefly, the robotic liquid handling system Genesis ProTeam 150 Advanced Digest (Tecan) was used to perform the tryptic in-gel digest with prior reduction/carboxamidomethylation of the proteins and to subsequently prepare the extracted tryptic peptides for MALDI-TOF-MS on prestructured sample supports (AnchorChip, Bruker Daltonics) according to the thin layer affinity method. Using an Ultraflex I MALDI-TOF/TOF mass spectrometer (Bruker Daltonics), peptide mass fingerprint spectra were automatically acquired, post-processed, and subjected to database searches as described. To confirm these results and to cover proteins often difficult to identify by peptide mass fingerprinting such as proteins in mixtures, post-translationally modified proteins and small proteins, the mass spectrometer was operated in the MS/MS mode within the same automated analysis loop to record fragment ion spectra of up to four selected precursor ions in a result-dependent manner. Data base searches in the Swiss-Prot or NCBInr primary sequence databases restricted to the taxonomy *Homo sapiens* were performed using the Mascot Software 2.0 (Matrix Science) licensed in-house. Carboxamidomethylation of cysteines was specified as fixed and oxidation of methionines as variable modification. The monoisotopic mass tolerance was set to 100 ppm and one missed cleavage was allowed. Database searches of MS/MS data sets were performed as above with the fragment mass tolerance set to 0.7 Da. Only proteins represented by at least one peptide sequence above significance threshold in combination with the presence of at least four peptide masses assigned in the peptide mass fingerprint were considered as identified.

***RNA interference.*** All siRNAs were double-stranded Silencer™ siRNAs, purchased from Ambion (Catalogue #: 16708) with the following properties: Pre-designed, annealed, standard purity (column purified). SiRNAs for cyclophilin A (positive control) and non targeting siRNA (scrambled sequences=negative controls) were HPLC purified. Once reconstituted in nuclease-free water, the siRNA was ready to transfect and was used at a final concentration of 10-90 nM. To transiently knock down CRABP1, 30 nM siRNA was used. We combined three different siRNAs, directed against different exons (10 nM each) to obtain a strong knockdown. Cells were grown in normal growth medium in 10 cm plates until they reached 50% confluency. Then the growth medium was substituted with 5 ml OPTI-MEM I without serum and the cells were incubated for 1-3 hours. Two mixtures (mixture a and b) were prepared in parallel: Mixture a: 33 µl of the transfection agent siPORT Amine, were diluted in 517 µl of OPTI-MEM I without serum to reach a final volume of 550 µl. The solution was mixed well and incubated at room temperature for 15 minutes. Mixture b: 10-90 nM siRNA was diluted into OPTI-MEM I to reach a final volume of 550 µl. The solution was incubated for 15 minutes at room temperature. The diluted siRNA (mixture b) was added to the diluted transfection agent (mixture a) and gently mixed. This mixture was incubated for 15 minutes at room temperature. 1.265 ml OPTI-MEM I were added to this mixture. After removing the OPTI-MEM I from the cells, the transfection agent/siRNA complex in OPTI-MEM I was added to the cells. The cells were incubated under normal cell culture conditions for 24 h. After 24 h, the medium containing the transfection agent/siRNA complex was substituted with normal growth medium and the cells were incubated for further 24 h. The total RNA was extracted from the cells 48 hours after transfection-start and the knock down of the gene of interest was measured by real-time PCR.

***Data analysis.*** Data analysis of genomic data was performed with the Microarray Suite MAS 5.0 (Affymetrix), Gene Operating Software GCOS 1.2 (Affymetrix), Array Assist 3.3 (Stratagene) and Excel (Microsoft). Cel-files, derived from MAS 5.0 and GCOS 1.2 were integrated into Array Assist and chp-files were created by using the PLIER algorithm. P-values were calculated from 3 independent experiments (corresponding to 3 single cell clones for C99WT, C99I45F, C99V50F and mock-contol) using either a two class unpaired t-test or analysis of variance (One Way ANOVA). Further filtering and sorting of data was performed with Excel.

***Differentiation assay.*** All preparation steps were carried out under dim light due to light-sensitiveness of RA. A 0.5 mM stock solution of RA in DMSO (both Sigma) was prepared. To reach final concentrations of 0.1 nM-1000 nM, this stock solution was further diluted in appropriate volumes of cell culture medium in absence or presence of serum. The human neuroblastoma cell line SH-SY5Y, grown on glass coverslips in 24 well plates, was treated with 0.1 nM-1000 nM in cell culture medium, in absence or presence of serum for 2-10 days. Differentiation was evaluated by checking the length, shape and number of outgrowing protrusions by phase contrast microscopy at appropriate times.

***Phase contrast microscopy.*** For evaluating differentiation after RA treatment, the human neuroblastoma cell line SH-SY5Y was grown on Neuroclean® Coverslips (Primeglass, Neuherberg, Germany) coated with collagen, which were placed into a 24-well plate. Differentiation was evaluated by the number, length and shape of protrusions. Phase contrast pictures were taken from living neuroblastoma cells. The Zeiss Axiovert 35 inverse photo-microscope, equipped with an Olympus DP50 digital colour camera, was used. Afterwards cells were again incubated at 37°C to induce further differentiation.

**5. Quality control of cells, target-RNA and arrays**

Each sample was screened and found to be free from mycoplasma contamination. The 260 nm/280 nm ratio for total-RNA was between 1.9-2.1 for chip experiments and 1.8-2.1 for quantitative real-time PCR. Total-RNA and unfragmented cRNA was checked with a Bioanalyzer 2100™. For total-RNA, two distinct bands (28s and 18s ribosomal RNA) were detected with the 28 s band approximately twice as strong as the 18 s band. For unfragmented cRNA a distribution of bands, representing the different mRNAs, was detected with an accumulation in the centre of each lane. For fragmented cRNA, bands, corresponding to a size of 35-200 bases, were detected. After scanning, array images were assessed by eye to confirm the absence of bubbles or scratches. The means of all chips are shown; the highest and lowest value is indicated in brackets. Target intensities of 100 (HG-U133 A Chips) and 20 (HG-U133 B Chips) were used. Only chips with equal target intensities were compared among each other (using Affymetrix MAS 5.0 array analysis software and Gene Chip operating software GCOS), scaling factors for A Chips were within acceptable limits 0.91 (0.8-1.4), as were background 75.1 (60.7-97.7), noise (rawQ) 2.7 (2.4-3.3) and number of present transcripts 51% (47.4-52.9%). 3'/5' ratios for *GAPDH* and β-actin were confirmed to be within acceptable limits (*GAPDH*: 0.92 (0.79-1.81), β-actin: 1.26 (1.03-2.29), and BioB spike controls were found to be present on 100% of all the chips, with BioC, BioD and CreX also present in increasing intensity. Scaling factors for all B Chips were within acceptable limits 1.24 (0.9-1.6), as were background 63.87 (43.9-112), noise (raw Q) 2.6 (2.0-3.6) and number of present transcripts 30% (14.3-38.4%). 3'/5' ratios for *GAPDH* and β-actin were confirmed to be within acceptable limits (*GAPDH*: 1.1 (0.88-2.03), β-actin: 1.3 (0.92-2.95), and BioB spike controls were found to be present on 95% of all the chips, with BioC, BioD and CreX also present in increasing intensity.

**6. RNA-quality assessed by using the Agilent 2100 Bioanalyzer**

**A**

**B**

Figure 6 Total-RNA (A) and unfragmented cRNA (B) were checked with a Bioanalyzer 2100™ (Agilent). The Bioanalyzer generates an artificial gel and electropherograms, providing information about RNA quality. A) For total-RNA, two distinct bands (28s and 18s ribosomal RNA) were detected with the 28 s band approximately twice as strong as the 18 s band. The two distinct bands appear slightly shifted from lane to lane (compared to the ladder) due to an unfixed Bioanalyzer or software problem but does not affect the quality of information. B) For unfragmented cRNA a distribution of bands, representing the different mRNAs, was detected with an accumulation in the center of each lane. Notice the wide range of size distribution in each lane showing unfragmented cRNA and the characteristic curves of the electropherograms. Only samples satisfying highest quality standards (a selection of such samples is shown here) were used for analysis.
